# Polyamide 11/Poly(butylene succinate) Bio-Based Polymer Blends

**DOI:** 10.3390/ma12172833

**Published:** 2019-09-03

**Authors:** Maria Laura Di Lorenzo, Alessandra Longo, René Androsch

**Affiliations:** 1 Institute of Polymers, Composites and Biomaterials (CNR), Via Campi Flegrei, 34, 80078 Pozzuoli (NA), Italy; 2 Department of Chemical, Materials and Production Engineering, University of Naples “Federico II”, P.le Tecchio 80, 80125 Napoli, Italy; 3 Interdisciplinary Center for Transfer-oriented Research in Natural Sciences, Martin Luther University Halle-Wittenberg, D-06099 Halle/Saale, Germany

**Keywords:** polyamide 11, poly(butylene succinate), polymer blends, bio-based polymers, biodegradable polymers, mechanical properties, thermal analysis, morphology, spectroscopy

## Abstract

The manuscript details the preparation and characterization of binary blends of polyamide 11 (PA 11) and poly(butylene succinate) (PBS), with PA 11 as the major component. The blends are fully bio-based, since both components are produced from renewable resources. In addition, PBS is also biodegradable and compostable, contrarily to PA 11. In the analyzed composition range (up to 40 m% PBS), the two polymers are not miscible, and the blends display two separate glass transitions. The PA 11/PBS blends exhibit a droplet-matrix morphology, with uniform dispersion within the matrix, and some interfacial adhesion between the matrix and the dispersed droplets. Infrared spectroscopy indicates the possible interaction between the hydrogens of the amide groups of PA 11 chains and the carbonyl groups of PBS, which provides the compatibilization of the components. The analyzed blends show mechanical properties that are comparable to neat PA 11, with the benefit of reduced material costs attained by addition of biodegradable PBS.

## 1. Introduction

Nowadays, synthetic polymers have replaced many traditional materials, such as wood, stone, metal, and ceramics, and they are used in all areas of daily life and application: they protect food and prevent spoilage, insulate electric cables, save fuel by making cars lighter and safer, are used as fabric for clothing, in contact lenses, etc. Out of about the 335 million tons of plastics produced annually, 99% are produced from petroleum [1], and it is expected that, by 2050, the plastics industry will account for 20% of the total oil consumed annually [2]. Such large use of synthetic polymers resulted in increasing environmental concern, which, coupled to the realization that petroleum resources are finite, led to considerable search for alternative sources of raw materials for the production of new polymers. Moving away from petrochemical feedstocks toward short-term renewable resources will moderate the detrimental influence of plastics on the environment. It is expected that the polymers that are produced from short-term, e.g., annually renewable feedstocks, which are often addressed as bio-based polymers, might fully replace those produced from fossil sources [3].

At present, several bio-based polymers are industrially produced, with applications that mainly include food packaging and agriculture, as well as biomedical devices [4,5,6]. Properties and the performance/cost ratio of bio-based polymers need to be improved in order to expand the variety of potential fields and enter new markets. A popular route applied for decades, also for classical polymers, is to tailor the properties of a given polymer for specific applications by blending [7,8,9]. This approach of enhancing properties is relatively inexpensive in comparison to developing new polymerization routes to attain novel polymers. In other words, research and development of new polymer blends play a crucial role in increasing the competitiveness of bio-based polymers.

One of the most important bio-based polymers is polyamide 11 (PA 11), which is a commercial aliphatic polyamide that is produced from castor oil [2,10,11]. Even if only representing a small fraction of the worldwide polyamide production, PA 11 finds applications in a wide range of fields, thanks to its biocompatibility, good oil and salt water resistance, excellent piezoelectric and cryogenic properties, and lower hydrophilicity as compared to the more widely used polyamides 6 and 6.6 [11,12]. Therefore, PA11 is used as an engineering polymer in a large range of industries, including automotive, offshore applications, as well as food packaging [13,14].

Many research efforts have been devoted to improve the properties of PA 11, mainly through incorporation of inorganic fillers [12,13,14,15,16,17,18], or by blending with other, especially bio-based polymers, like poly(l-lactic acid), or polyhydroxyalcanoates to further expand the application range [19,20]. Among the various bio-based polymers, blends of PA 11 with poly(butylene succinate) (PBS) have barely been investigated. To our knowledge, only a single study about PA 11/PBS blends was performed, where a composition-dependent improvement of the impact strength of PA 11 was reported, without affecting other mechanical properties, like the flexural modulus [21]. From these results, it appears that the addition of PBS has potential to improve properties of PA 11, also taking into account that PBS is not only bio-based, but also biodegradable and compostable [22,23], contrarily to PA 11. In addition, the production of PBS only requires half the costs as the production of an equivalent amount of PA 11 [24,25,26]. Therefore, PA 11/PBS blends appear as promising new polymeric materials, with reduced material/production costs as compared to plain PA 11, and expected to be also partially biodegradable.

The biodegradation of PBS chains is initiated by the hydrolysis of ester bonds, which leads to the formation of water-soluble fragments with a molar mass lower than 500 Da. These short PBS chain segments can be assimilated by microorganisms, and then turned to carbon dioxide, water, and biomass [22,23]. Conversely, PA 11 is not easily decomposable in the environment. Hence, PA 11 formulations containing PBS may be partially biodegradable, with the extent of biodegradation of these blends currently under investigation.

PA 11 and PBS are both semi-crystalline polymers. PA 11 is rigid at room temperature, with a glass transition temperature of the mobile amorphous fraction (MAF) of *T*_g_ = 43 °C [27], and a rigid amorphous fraction (RAF) that devitrifies at higher temperatures [28]. It also exhibits crystal polymorphism that depends on thermo-mechanical history, with six different crystal modifications being reported in the literature [29,30,31,32]. After melt processing, like injection-molding [33], the polymer is semi-crystalline at room temperature, often containing lamellar crystals, which were grown to form a spherulitic superstructure. The maximum crystal fraction, similar as in case of other linear polyamides, is well below 50%, however, contributing to its balanced property profile [27]. Further dedicated studies about semi-crystalline morphologies of PA 11 forming, e.g., at high supercooling of the melt, or developed upon self-nucleation are available in the literature [34,35]. At room temperature, PBS has a rubbery mobile amorphous fraction, with a glass transition below −30 °C [36], as well as sizable RAF, whose vitrification/devitrification was quantified in [37] as a function of the cooling rate. In addition, the melting points of the two polymers largely differ, with the equilibrium melting temperature ($T_{m}^{o}$) of PBS in the range of 127.5–146.5 °C [38], whereas PA 11 has a $T_{m}^{o}$ = 203–220 °C [39,40].

PBS is already used as additive in other bio-based polymer formulations, for instance, in blends with poly(lactic acid) (PLA), to improve the flexibility, toughness, and heat resistance of PLA [41], and it is likely that the addition of PBS also has potential for improving properties of PA 11. As mentioned above, PBS may improve the impact strength of PA 11 while maintaining the flexural modulus, as recently published in a short communication [21]. However, information regarding these blends are limited to quantitative data on impact strength and modulus, supported by analysis of morphology of the blends [21]. The improvement of mechanical properties of PA 11 upon the addition of PBS, as probed in [21], deserves a more detailed investigation, to fully exploit the potential of these blends and optimize both composition and processing. For these reasons, a detailed investigation of the influence of PBS on the thermal and mechanical properties of PA 11 is needed, which is reported in this manuscript for blends containing the polyamide as the main component. This will be completed by a thorough analysis of the influence of thermal history on the crystallization kinetics of both polymers, to be presented in a forthcoming manuscript.

## 2. Experimental Part

### 2.1. Materials

A heat- and light-stabilized PA 11, extrusion grade Rilsan^®^ BESNO TL from Arkema (Colombes, France), was used. The melt volume index of the polymer is 1 cm^3^/10 min (235 °C, 2.16 kg) [42] and the number-average molar mass and polydispersity are 17.2 kg mol^−1^ and around 2, respectively [35]. Further material data are available at the Arkema website [42].

PBS Bionolle 1001MD was kindly received by Showa Denko K. K. (Japan). This polymer grade has a melt-flow index of 1.4 g/10 min (190 °C, 2.16 kg). The number-average molar mass and polydispersity are 57.7 kg mol^−1^ and 2, respectively, as measured by gel permeation chromatography.

Before melt mixing, the PA 11 and PBS pellets were dried in a vacuum oven at 80 °C for 4 h, or 60 °C for 16 h, respectively.

### 2.2. Blend Preparation

Binary PA 11/PBS blends with a composition of 100/0, 90/10, 80/20, 60/40 m/m% were prepared by melt mixing in a Brabender-like apparatus Rheocord EC of Haake Inc. (Vreden, Germany) at 210 °C and 32 rpm for 8 min.

### 2.3. Preparation of Compression-Molded Sheets

PA 11/PBS blends were compression-molded with a Collin Hydraulic Laboratory Forming Press P 200 E. The blends were heated to 210 °C and then kept at this temperature for 2 min without application of any pressure, to allow for complete melting. After this period, a load of 0.5 tons was applied for 2 min, and then the sample was cooled to room temperature in less than 3 min by means of cold water circulating in the plates of the press. Compression-molded sheets with a thickness of about 300 µm were obtained. A compression-molded sheet of plain PBS was also prepared, by pre-melting at 135 °C for 2 min, followed by molding with a load of 0.5 ton for 2 min, and then cooling to room temperature via cold water circulating in the plates of the press. A lower molding temperature was used to attain sheets with the same thickness as the PA11/PBS blends. However, the varied thermal history did not lead to significant variation in the analyzed material properties, which were limited to thermal stability (thermal degradation of PBS initiates above 300 °C, as shown below), glass transition, and crystallization/melting behavior (the cooling rate from the relaxed melt was the same as the blends), nor affected infrared spectroscopy analysis.

### 2.4. Differential Scanning Calorimetry

Differential scanning calorimetry (DSC) was performed with a Perkin-Elmer Pyris Diamond DSC (Waltham, MA, USA) that was equipped with an Intracooler II as cooling system. The instrument was calibrated in temperature and energy with a high purity indium standard, using dry nitrogen as purge gas at a flow rate of 30 mL min^−1^. The compression-molded blends were analyzed upon heating at 20 K min^−1^, from −60 to 210 °C. The molded samples had a thickness of 300 μm, which corresponds to about 6 mg when cut to fit into DSC sample pans. The experimentally measured heat-flow-rate raw data were corrected for instrumental asymmetry by subtraction of a baseline, measured under identical conditions as the samples, including a close match of the masses of the aluminum pans. All of the experiments were repeated three times to ensure reproducibility.

### 2.5. Thermogravimetry

Thermogravimetric analyses (TGA) were carried out with a Perkin Elmer Pyris Diamond TG-DTA instrument under nitrogen atmosphere. Measurements were performed on samples of about 5 mg, and then heated from room temperature to 600 °C at 10 K min^−1^ in nitrogen atmosphere, with a nominal gas flow rate of 30 mL min^−1^. 

### 2.6. Fourier-Transform Infrared Spectroscopy

Fourier-transform infrared spectroscopy (FTIR) spectroscopy was performed in reflection mode while using a PerkinElmer FTIR Spectrometer Model Spectrum 100 equipped with a PerkinElmer Universal Attenuated Total Reflectance (ATR) sampling accessory with a diamond crystal. Each spectrum is an average of 16 individual scans, recorded at a resolution of 2 cm^−1^. The compression-molded blends were analyzed by directly placing the films on the diamond crystal. Each spectrum was repeated three times.

### 2.7. Scanning Electron Microscopy

Morphological analysis of cryogenically fractured PA 11/PBS blends was performed while using a FEI Quanta 200 FEG environmental scanning electron microscope (ESEM) (Eindhoven, The Netherlands) in low vacuum mode, while using a Large Field Detector (LFD) and an accelerating voltage of 30 kV. Before analysis, the samples were sputtered-coated with an Au–Pd alloy using a Baltech Med 020 Sputter Coater System and mounted on aluminum stubs by means of carbon adhesive disks.

### 2.8. Tensile Tests

Dumbbell-shaped specimens with a length and thickness of the gauge section of 25 and 4 mm, respectively, were cut from the compression molded sheets and then used for tensile measurements. Stress-strain curves were obtained with an Instron machine, Model 4505 (Norwood, MA, USA) at a cross-head speed of 5 mm min^−1^. Young’s modulus, stress, and strain at yield and at break were calculated from an average of seven specimens.

## 3. Results and Initial Discussion

**Figure 1:**
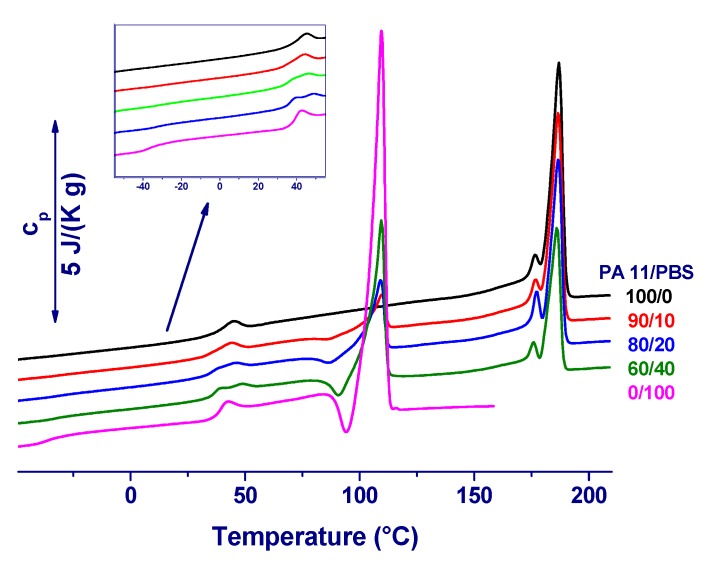
Apparent specific heat capacity (*c*_p_) of compression-molded PA 11/PBS sheets as a function of temperature, measured upon heating at 20 K min^−1^. Data are shifted vertically for clarity.

Figure 1 illustrates the apparent specific heat capacity (*c*_p_) plots of PA 11/PBS compression-molded sheets, as measured upon heating at 20 K min^−1^, one day after preparation. Plain PA 11 (black curve) displays a glass transition temperature (*T*_g_) centered at 41 °C, as typical for PA 11 [27,39], with the corresponding heat-capacity increment overlapping with a small enthalpy-recovery peak that is caused by the short storage at room temperature (i.e., below *T*_g_), as well as by the different cooling/heating rates used [43]. The material shows double melting due to crystal reorganization on slow heating, which is in agreement with literature data [27]; the final melting peak is detected at 188 °C.

The DSC plot of plain PBS (magenta curve) shows a *T*_g_ of −36 °C, with a *c*_p_-jump that points to a mobile amorphous content of *w*_A_ = 0.22, as calculated by comparison the measured *c*_p_-step to the heat capacity step at *T*_g_ of the fully amorphous polymer [37,44]. This is followed by a small endotherm peaked at 42 °C, coupled with a sizable increase of *c*_p_, and then by a broad endotherm, a sharp recrystallization, and a final melting peak. The overall crystal fraction, as measured by a comparison of the experimentally observed enthalpy of fusion and the heat of fusion of 100% crystalline PBS of 220 J g^−1^ [45] indicates a crystal fraction *w*_C_ = 0.28. The rigid amorphous fraction, *w*_RA_, as calculated by difference [46,47,48], amounts to *w*_RA_ = 0.50. Small endotherms at temperatures close to 40 °C have been reported in the literature for PBS, and ascribed to the melting of small crystals that is caused by annealing at temperatures above *T*_g_ [45]. However, the integration of the endotherm peaked at 42 °C in Figure 1 leads to a small enthalpy of transition that may be linked to melting of only 1% of PBS crystals, a too low amount to justify the marked increase in *c*_p_, which instead reveals the mobilization of 0.35 of solid fraction. It may be speculated that the endotherm at 42 °C is linked to enthalpy relaxation of the RAF, which partly mobilizes in this temperature range [37].

The DSC plots of PA 11/PBS blends containing between 10 and 40 m% PBS display multiple thermal events, which are close to the algebraic sum of the *c*_p_ curves of the two polymers. The glass transition of PBS, centered at −36 °C [36,49], is hardly detectable in the 90/10 blend, but it becomes more intense with the increasing content of PBS in the blend, as seen in the insert of Figure 1. *T*_g_ of PA 11 overlaps with the small endotherm peaked at 42 °C in plain PBS, with the latter intensifying with a higher PBS content. When compared to neat polymers, the melting of PA 11 crystals in the blends seems scaling in size with composition, whereas the recrystallization and melting of PBS crystals in the blends also display a small shift in temperature. The detection of two separate *T*_g_’s in the blends, occurring at the same temperatures as in the plain polymers, indicates the immiscibility of PA 11 and PBS. This is confirmed by the occurrence of separate crystallization and melting events of the two polymers, but with some influence of PA 11 on crystallization and melting of PBS chains.

The crystal fractions of PA 11 and PBS in the blends were determined by the integration of the melting endotherms and normalization to the content of the respective blend component. The measured melting enthalpy of PA 11 fraction is 40 ± 1 J g^−1^, independent of the blend composition. PA 11 is a polymorphic polymer and it exhibits six different crystalline phases [29,30,31]. The melting profile of PA 11 that is shown in Figure 1 indicates presence of the α-modification, as it is expected for the selected pathway of preparation [27]. Literature data on the bulk enthalpy of melting of PA 11 α-crystals vary from 189 to 244 J g^−1^ [27], which leads to a crystal fraction of the PA 11 part (*w*_C,PA_) of 0.16 to 0.21, respectively. Additionally, the heat of fusion of the PBS fraction, normalized to its content in the blends, seems to be unaffected by the blend composition, and it amounts to 55 ± 0.5 J g^−1^. A comparison of the measured heat of fusion with the heat of fusion of 100 % crystalline PBS of 220 J g^−1^ [45] results in a crystal fraction of the PBS parts in the blends (*w*_C,PBS_) of 0.26. As such, the total crystal fraction (*w*_C_ = *w*_C,PA_ + *w*_C,PBS_) of the compression molded sheets linearly increases with the addition of PBS, due to its larger crystallinity, from 16% in plain PA 11 to 20% in the PA 11/PBS blend 60/40.

**Figure 2:**
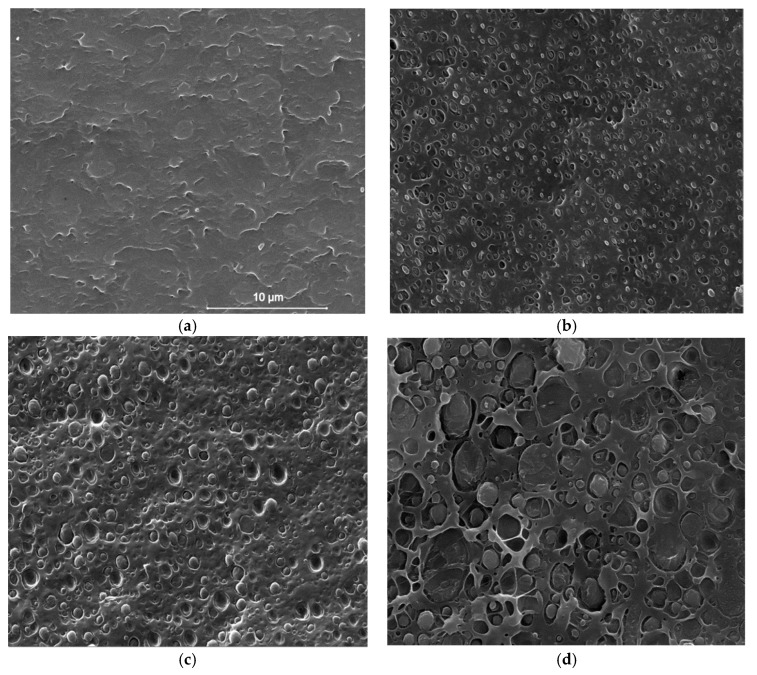
Scanning electron micrographs of cryogenically fractured surfaces of PA 11/PBS blends: (**a**) 100/0; (**b**) 90/10; (**c**) 80/20; and, (**d**) 60/40.

To gain information about the phase structure and morphology of PA 11/PBS blends, scanning electron microscopy (SEM) analyses were performed, with the results presented in Figure 2. Figure 2a illustrates the cryogenically-fractured surfaces of compression-molded, plain PA 11, which appears smooth, as expected. The addition of PBS (Figure 2b–d) results in formation of a matrix-particle structure, that is, there occurred phase separation. The size of the particles increases with PBS content in the blends, which, coupled to the small voids surrounding the minor phase inclusions, seems to suggest a scarce interfacial adhesion. Otherwise, the PBS particles appear homogeneously distributed within the matrix, and most of them were not pulled out during the cryogenical fracture process, rather than remained attached to the matrix, perhaps indicating compatibility between the blend components.

**Figure 3:**
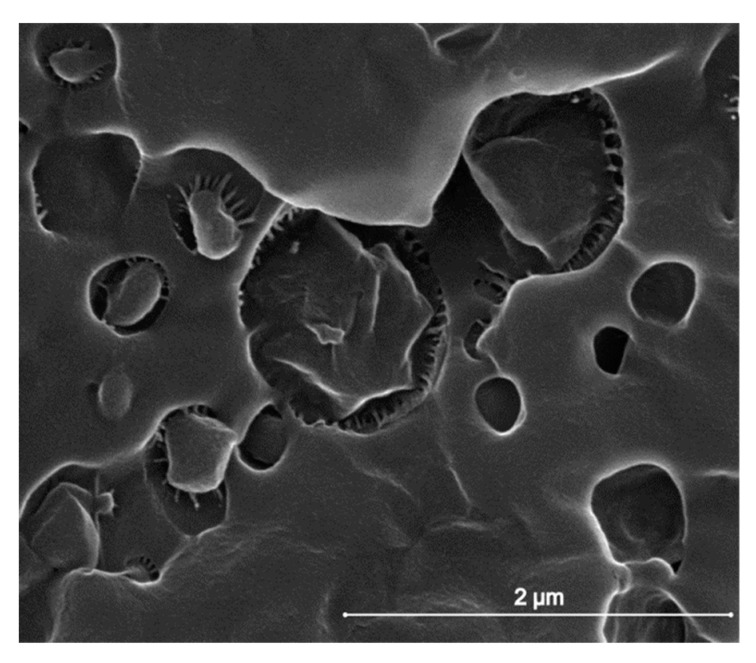
Scanning electron micrograph of a cryogenically fractured surface of the PA 11/PBS 60/40 blend.

Close inspection of the cryogenically fractured surfaces revealed the presence of small fibrils between the dispersed PBS particles and the PA 11 matrix. This is illustrated in Figure 3, which shows the morphology of the cryogenically fractured 60/40 blend at higher magnification. PBS droplets appear to be bonded to PA 11 via small fibrils, which join the two phases, however leaving some voids. Such fibrils are observed in all of the analyzed blends of different composition, and they are also reported in [21]. These fibrils/voids may form during fracture, due to the release of residual/internal stress imposed to the blends by crystallization/volume contraction of the components at different temperatures, by different thermal shrinkage of the PA 11 and PBS, or by cooling-rate gradients during melt-processing [50]. In fact, the fibrils indicate coupling of the matrix and particles, which is perhaps due to interaction between the functional groups of PA 11 and PBS macromolecules, established during melt mixing, which might provide partial compatibilization of the blend components.

**Figure 4:**
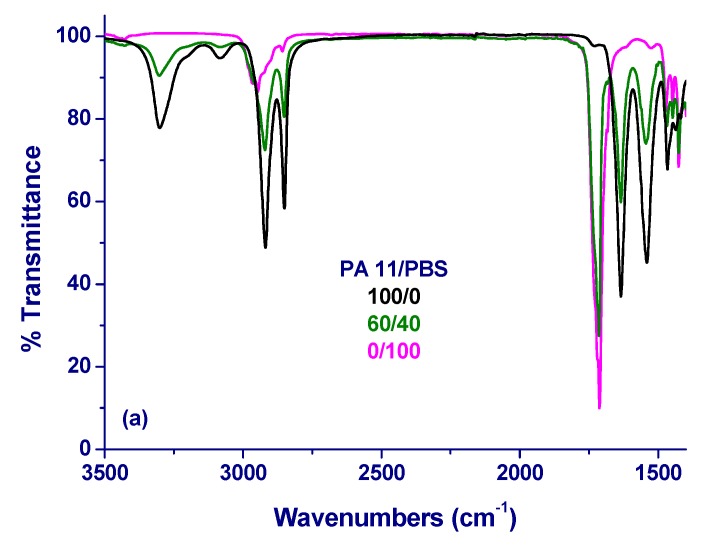

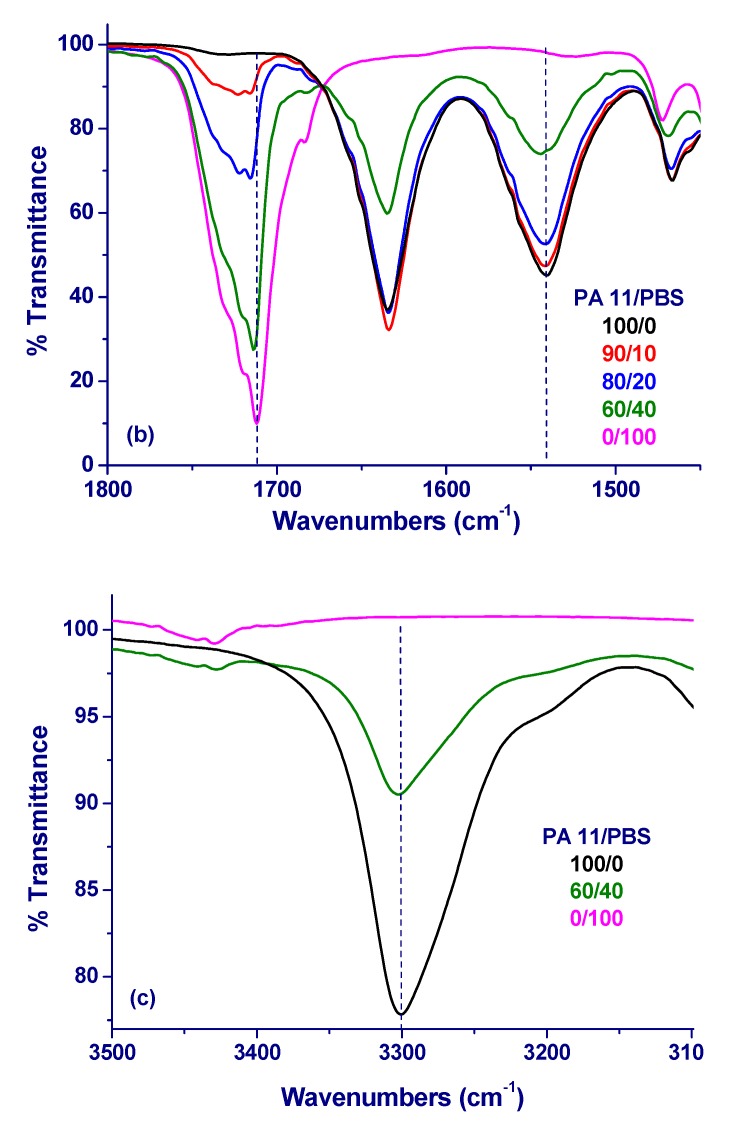
Fourier-transform infrared spectroscopy-Attenuated Total Reflectance (FTIR-ATR) spectra of compression-molded samples of PA 11/PBS blends: (**a**) plain polymers compared to the 60/40 blend; (**b**) the whole analyzed composition range, in the wavenumber range from 1800 to 1450 cm^−1^; and, (**c**) enlargement of the plots shown in (**a**) to highlight changes in the wavenumber range of stretching of N–H band of PA 11.

The type of interaction was investigated by FTIR-ATR analysis. Figure 4a shows the FTIR-ATR spectra of plain PA 11 (black), plain PBS (magenta), and the 60/40 blend (green) in the wavenumber range from 3500 to 1400 cm^−1^. The FTIR-ATR spectrum of plain PBS has a complex band at around 1712 cm^−1^, which is the convolution of three different C=O stretching modes at around 1736, 1720, and 1714 cm^−1^ [51]. The band at 1736 cm^−1^ corresponds to the stretching mode of C=O in the mobile amorphous fraction, the one at 1720 cm^−1^ is assigned to the stretching in the rigid amorphous fraction and the one at 1714 cm^−1^ regards the crystalline phase. The FTIR-ATR spectrum of PA 11 has a band at 1635 cm^−1^ that corresponds to the stretching mode of C=O, a band at 3300 cm^−1^ that is assigned to the N–H stretching and the amide II band at 1540 cm^−1^. This latter band results from an interaction between N–H bending and the C–N stretching of the C–N–H group.

Focusing on the FTIR-ATR spectra of the blends in the range between 1450 and 1800 cm^−1^ (Figure 4b), the C=O stretching band in plain PBS shows a shift of 3 cm^−1^ on the addition of 10 m% PA 11. At the same time, a shift of the N–H stretching mode band and the amide II band of PA11 is observed. The amide II band progressively shifts from 1540 cm^−1^ in plain PA 11 to 1544 cm^−1^ in the 60/40 blend, together with a shift of 2 cm^−1^ on the N–H stretching band at 3300 cm^−1^, as shown in Figure 4c.

These spectral changes can be rationalized while taking interaction between the carbonyl group of PBS and the N–H group of the amide in PA 11, established upon melt mixing, into account. Even if the shifts are low in terms of frequencies, those are reproducible, hence not are connected to instrumental errors. With the available experimental data, such small variations in wavenumbers can only be ascribed to hydrogen bonding, while also taking into account that the shift of the C=O stretching band of PBS is too low to be explained with a change in the crystalline structure of the polymer. However, the latter was shown not to vary by DSC analyses of Figure 1. 

**Figure 5:**
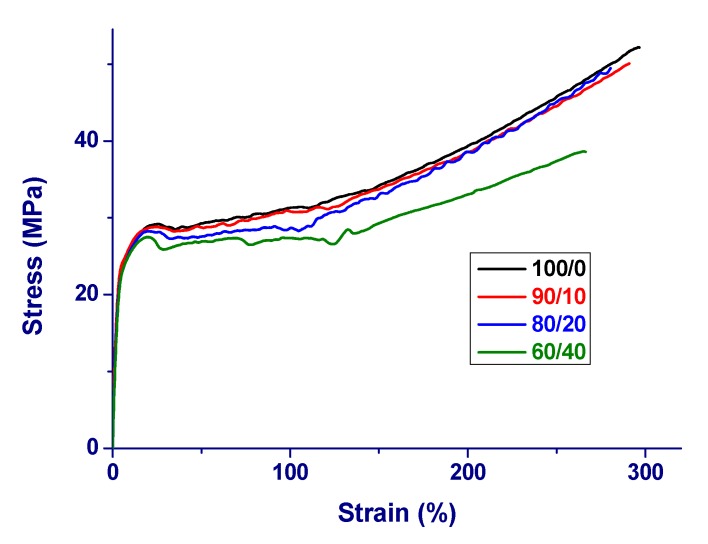
Engineering tensile stress-strain plot of PA 11/PBS blends, measured at room temperature, with a crosshead speed 5 mm min^−1^.

The nominal stress-strain curves of PA 11 and PA 11/PBS blends that were obtained at room temperature are presented in Figure 5. Table 1 summarizes the Young’s modulus (*E*), yield stress and strain, and stress and strain at break. All of the analyzed materials show ductile behavior. The deformation of plain polyamide occurs in three stages, as typical for semi-crystalline polymers [52,53,54]: a first stage, where the stress sharply increases with strain, followed by a homogeneous plastic deformation stage, then a necking phenomenon occurs, which shows that the deformation becomes heterogeneous, until the sample breaks. Plain PA 11 displays a Young’s modulus of *E* = 930 MPa, yielding occurs at strain ε_y_ = 25%, and stress σ_y_ = 29 MPa, and rupture at elongation ε_r_ = 300%, and stress σ_r_ = 52 MPa. The measured parameters are in line with literature data [15,55,56]. The addition of PBS results in a small variation of the measured mechanical parameters, with a small progressive decrease of the Young’s modulus with the addition of PBS, as well as of the yielding and break parameters.

Literature data of Young’s modulus of plain PBS range from 500 to 590 MPa [57,58], i.e., the modulus is lower than in PA 11. PBS is more ductile than PA 11, with ε_r_ = 950% [59], although some authors also reported PBS as being a brittle polymer [60,61], probably due to material degradation [62]. All of the analyzed PA 11/PBS blends display ductile behavior, exhibiting elongation values that resemble those of the plain polyamide.

It is well known that in semi-crystalline polymers Young’s modulus increases with the crystal fraction [63,64,65]. The slight decrease of Young’s modulus in the blends is caused by the lower modulus of PBS, which overwhelms the somewhat higher overall crystallinity of the material. Being measured at low strain, Young’s modulus is not significantly affected by the blend morphology, whereas large-strain properties, like stress and strain at break, strongly depend on the morphology, including phase separation in the amorphous phase, as well as the homogeneity of dispersion and adhesion between the phases. Good tensile properties, such as tensile strength and yield and ultimate parameters, can be expected if the domains are small in size and well dispersed in the surrounding matrix [66]. As seen in Figure 2, the PA 11/PBS blends present an island-matrix morphology with PBS particles surrounded by small voids, and with a particle size that varies with blend composition. However, the PBS particles are not decoupled from the matrix, but are kept connected with fibrils, which is probably due to interaction between the amide and carbonyl groups of the two polymers, as suggested by FTIR-ATR analysis. Such fibrils that bridge the PA 11 and PBS phases allow the samples to bear high elongation, comparable to that of pure PA 11, with a small decrease only observed in the blend containing 40 m% of the dispersed component.

**Figure 6:**
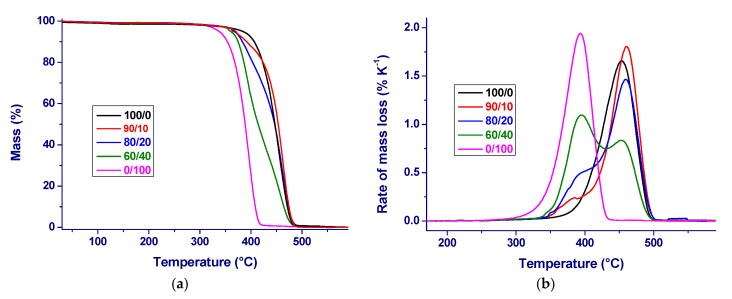
Thermogravimetry (TGA) plots of PA 11/PBS blends, measured in nitrogen atmosphere upon heating at 10 K min^−1^: (**a**) normalized mass as function of temperature; and, (**b**) rate of mass loss as function of temperature.

The influence of PBS on the thermal stability of PA 11 was investigated by thermogravimetry (TGA). The TGA plot of PA 11/PBS blends, analyzed upon heating at 10 K min^−1^ in nitrogen atmosphere, is presented in Figure 6a, while the derivative thermogravimetry curves are shown in Figure 6b. The TGA curve of plain polyamide displays a slow mass loss between 200 and 380 °C, with an overall mass decrease of about 5%. A further increase of the temperature leads to a steep decrease of the polymer mass, with full decomposition of the polymer just below 500 °C [25], and a maximum degradation rate being observed at 455 °C, as seen in Figure 6b. Pure PBS also undergoes thermal degradation in a single step, with no significant mass loss until about 300 °C and complete decomposition around 430 °C, which is in agreement with the literature data [67,68]. The primary mass loss is caused by the volatilization of small molecules, including succinic acid and butylene glycol, followed by major thermal degradation of PBS chains, due to random chain scission at the ester bonds, with the formation of carboxylic end groups and vinyl groups [38].

In the blends, thermal degradation is initiated at the same temperature as the degradation of plain PBS, with all of the analyzed compositions displaying two major steps of mass loss, mostly resembling those of the pure components. The peaks in the degradation rate of the PBS particles of the blends appear unchanged as compared to the pure polymer, with a minor shift of the degradation of the PA 11 portions of the blends, which undergo degradation at slightly higher temperatures. This suggests some minor influence of the degradation products of PBS on the thermal degradation of PA 11.

## 4. Final Discussion and Conclusions

Previous research indicated that PBS can be used to improve mechanical properties of PA 11, specifically the impact strength. These data are now completed by a full analysis of the blend morphology, the phase structure, thermal, and tensile properties of PA 11/PBS blends, with PA 11 as major component.

In the analyzed composition range from 0 to 40 m% PBS, PA 11 and PBS are immiscible and form a four-phase morphology made of two crystal phases, plus two amorphous phases. The components arrange in a particle-matrix morphology, with roughly spherical PBS particles that are suspended in the PA 11 matrix and small voids surrounding the minority-phase inclusions. Increasing the PBS content from 10 to 40 m% results in an increase of the average particle size. Despite the immiscibility of the blend components, the matrix and particles display interfacial adhesion, as most PBS particles are not removed upon cryogenical fracture, but they remain anchored to the matrix. Interaction between the amide groups of PA 11 chains and the carbonyl groups of PBS was proven by FTIR-ATR analysis, which may rationalize the adhesion between the phases. This is revealed also by the SEM micrographs of the fractured surfaces of the blend that display small fibrils that bridge the PBS inclusions with the PA 11 matrix.

It may be advisable, and the subject of further investigation, to investigate the effect of addition of a compatibilizer that may further improve the interfacial adhesion between the phases. At the level of compatibility that is only attained by melt blending PA 11 and PBS, the PA 11-rich binary blends display mechanical properties that are comparable to neat PA 11. There is only observed minor variation of the Young’s modulus and of the yielding and break parameters, despite that PBS alone can sustain much higher elongation, up to 900% of the initial value, when compared to the 300% typical of PA 11 and of the analyzed blends. In other words, it is likely that further increase of interfacial adhesion, which may be provided by a compatibilizer, or by further promoting reaction between the functional groups of the two polymers, might lead to a blend formulation with improved mechanical properties as compared to pure PA 11, and that is also partially biodegradable.

The analysis of the thermal properties indicated little influence of the blend composition on thermal degradation, with only a minor shift of the degradation temperature of the PA 11 matrix upon the addition of PBS. The amorphous phases have separate glass transitions whose temperature seems not be affected by blending, at least for the analyzed composition range. Blending seems not to influence the crystallization of PBS and PA 11, which might appear in contrast with the reported interaction between PA 11 and PBS phases. However, the data that are presented in this manuscript are limited to the processing history imparted upon melt-mixing using a specific extruder and compression molding. The condition of crystallization of PA 11 can influence crystallization kinetics of PBS parts of the blends, which may help in modulating the crystallinity and crystal morphology, and, in turn, material properties, as will be detained in a forthcoming manuscript that illustrates a thorough analysis of crystallization kinetics of both polymers in the blends.

## Figures and Tables

**Table 1:** Tensile parameters of PA 11/PBS blends.

PA 11/PBS	E (MPa)	σ_y_ (MPa)	ε_y_ (%)	σ_r_ (MPa)	ε_r_ (%)
100/0	930 ± 40	29 ± 1	25 ± 2	52 ± 3	300 ± 20
90/10	910 ± 30	29 ± 1	25 ± 3	50 ± 3	290 ± 20
80/20	890 ± 40	28 ± 2	23 ± 5	49 ± 5	280 ± 20
60/40	870 ± 30	27 ± 2	20 ± 3	39 ± 5	270 ± 30
